# Epiphytic bacterial community composition on four submerged macrophytes in different regions of Taihu Lake

**DOI:** 10.3389/fpls.2024.1404718

**Published:** 2024-07-25

**Authors:** Hongda Fang, Zhuo Zhen, Fan Yang, Hailei Su, Yuan Wei

**Affiliations:** ^1^ College of Harbour and Coastal Engineering, Jimei University, Xiamen, China; ^2^ Key Laboratory of Urban Environment and Health, Institute of Urban Environment, Chinese Academy of Sciences, Xiamen, China; ^3^ State Key Laboratory of Environmental Criteria and Risk Assessment, Chinese Research Academy of Environmental Sciences, Beijing, China

**Keywords:** epiphytic bacteria, submerged macrophyte, host-specificity, habitat heterogeneity, lottery model

## Abstract

The epiphytic bacteria in aquatic ecosystems, inhabiting a unique ecological niche with significant ecological function, have long been the subject of attention. Habitat characteristics and plant species are believed to be important in controlling the assembly of epiphytic bacteria. However, the underlying principle governing the assembly of the epiphytic bacterial community on macrophytes is far from clear. In this study, we systematically compared the diversity and community composition of epiphytic bacteria both in different habitats and on different species of macrophytes where they were attached. Results suggested that neither the plant species nor the habitat had a significant effect on the diversity and community of epiphytic bacteria independently, indicating that the epiphytic bacterial community composition was correlated to both geographical distance and individual species of macrophytes. Furthermore, almost all of the abundant taxa were shared between different lake regions or macrophyte species, and the most abundant bacteria belonged to Proteobacteria and Firmicutes. Our results demonstrated that the competitive lottery model may explain the pattern of epiphytic bacterial colonization of submerged macrophyte surfaces. This research could provide a new perspective for exploring plant–microbe interaction in aquatic systems and new evidence for the lottery model as the mechanism best explaining the assembly of epiphytic bacteria.

## 1 Introduction

Submerged macrophytes, providing a linkage between sediment and overlying water, play an important role in the biological productivity and stability of the structure and function of aquatic ecosystems (Horppila and Nurminen, 2003). They are also used as indicators of the ecological quality of lakes because they have a clear response to eutrophication (Søndergaard et al., 2010). Furthermore, submerged macrophytes with a large surface area enhanced the density of surface-associated organisms, such as algae, invertebrates, and bacteria (Jeppesen, 1998). In aquatic systems, a large number of bacteria, which were described as epiphytic bacteria in previous studies (Andrews and Harris, 2000; Pollard, 2010), inhabit surfaces of submerged macrophytes. According to previous studies, epiphytic bacteria exhibited a higher diversity and a distinct community composition compared to the surrounding bacterioplankton (He et al., 2014; Levi et al., 2017) and play important ecological roles in aquatic ecosystems (Ma et al., 2021). Moreover, epiphytic bacteria may also have complex interactions with their host plants. These interactions are manifested as competitive, mutualistic, and commensalistic (Wijewardene et al., 2022). Despite these findings, epiphytic biofilms are understudied compared to other periphytic biofilms in freshwater ecosystems (Wijewardene et al., 2022). Ignoring the understanding of the role macrophytes play as a substrate for epiphytic bacteria may underestimate the importance of macrophytes in freshwater ecosystems.

For decades, the relationship between epibiotic bacterial community and environmental factors (including host plants, spatial and geographical heterogeneity) and hypotheses regarding bacterial community assembly has been studied extensively (Knief et al., 2010; He et al., 2012; Liao et al., 2016; Levi et al., 2017). However, due to differences in research emphasis and technical matters, there were some inconsistencies and even contradictory conclusions in different scientific reports. Therefore, the relationship between bacterial composition and host plants/habitats, and the mechanisms of community assembly are still poorly understood. There are two theories explaining the mechanism of bacterial community assembly. One is the traditional niche-based theory, which emphasizes abiotic and biotic factors, such as environmental physicochemical properties, habitat heterogeneity, and species interactions (Ramette and Tiedje, 2007; Dumbrell et al., 2010; Gilbert et al., 2012). The other is the neutral theory that only considers random processes, such as birth, death, colonization, immigration, and speciation or dispersal limitations (Vanwonterghem et al., 2014). Recent investigations have suggested that both neutral and niche processes play a critical role in the assembly of entire bacterial communities (Liao et al., 2016). In the case of epiphytic bacteria, some studies demonstrated that the composition of the epiphytic bacterial community may be influenced by the host plant and habitat heterogeneity (Hempel et al., 2008), while other studies suggested that plant-specific effects could shape the composition of epiphytic bacteria on aquatic plants (He et al., 2012; Yu et al., 2022). These references provided valuable information about the complex interaction between epiphytic bacteria and their host plants. However, few studies have systematically investigated the influence of habitat heterogeneity and plant–host specificity on the community composition of epiphytic bacteria. Given that situation, we questioned whether different host plants or their respective habitat determined the epiphytic bacterial community composition.

In the study, we collected four species of submerged macrophytes (*Ceratophyllum demersum*, *Vallisneria natans*, *Myriophyllum verticillatum*, and *Potamogeton crispus*) from three regions of Taihu Lake with different trophic status. This work aimed to 1) ascertain whether macrophyte species or their habitats determine epiphytic bacterial community composition, 2) investigate the mechanisms of assembly for epiphytic bacteria, and 3) give directions to further research.

## 2 Materials and methods

### 2.1 Study sites and sample collection

**Figure 1:**
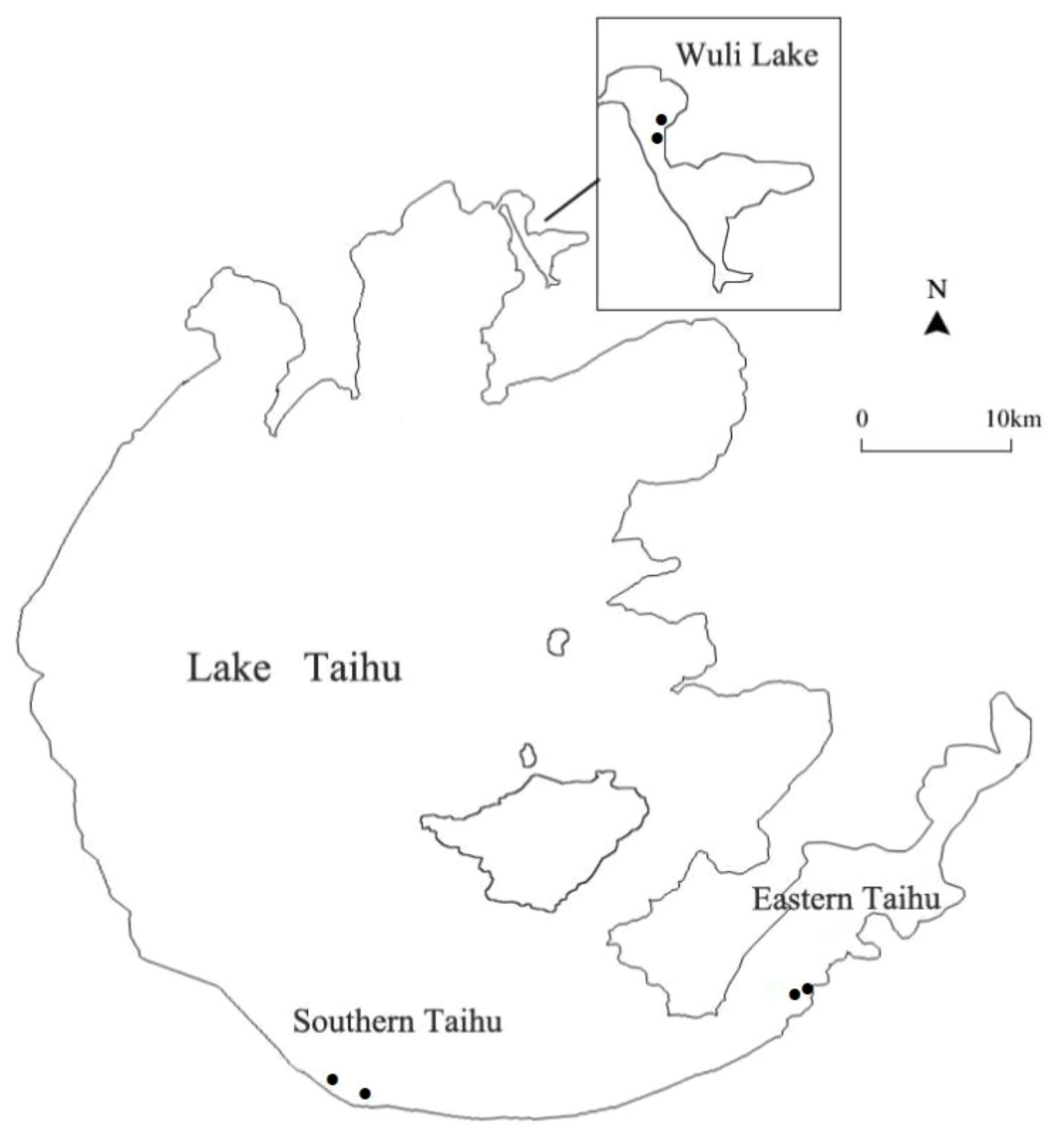
Map of Lake Taihu showing the sampling locations in Southern Taihu Lake, Eastern Taihu Lake, and Wuli Lake.

Taihu Lake, the third largest shallow freshwater lake (2,338 km^2^, average depth 1.9 m) in China, is located in the Yangtze River Delta (Qin et al., 2007). There are strong environmental gradients and habitat patterns in Taihu Lake (Dong et al., 2017). In this study, samples were collected from three regions of Taihu Lake: Wuli Lake (W), Southern Taihu (ST), and Eastern Taihu Lake (ET) (**Figure 1**). Wuli Lake, an arm of Meiliang Bay in the north of Taihu Lake, is a hyper-eutrophic bay located in Wuxi City. The Southern Taihu Lake features less embayment, little macrophyte coverage, and relatively good water quality and is classified as mesotrophic water. Eastern Taihu Lake, a shallow macrophyte-dominated bay and the area where the dilution water flows out of the lake (Hu et al., 2010), is generally considered the least nutrient-impacted region of Taihu Lake.

Four macrophytes were collected by hand or hook on July 24–26, 2016. For each plant species, three to five individual plants at a similar growth stage were collected randomly in three selected regions of Taihu Lake and Wuli Lake (**Table 1**). Plants were stored individually in sterile plastic bags on ice and transported to the laboratory. Water parameters such as pH, temperature, chlorophyll-*a*, and conductivity were measured using a multi-parameter water quality analyzer (YSI 6600v2) prior to sample collection (**Supplementary Table S1**).

**Table 1:** Plant species and sampling locations.

Site description	Plant species	Sample code
Eastern Taihu Lake	*Myriophyllum verticillatum*	ET-M
	*Vallisneria natans*	ET-V
	*Potamogeton crispus*	ET-P
Southern Taihu Lake	*Myriophyllum verticillatum*	ST-M
	*Vallisneria natans*	ST-V
	*Potamogeton crispus*	ST-P
	*Ceratophyllum demersum*	ST-C
Wuli Lake	*Myriophyllum verticillatum*	W-M
	*Vallisneria natans*	W-V
	*Ceratophyllum demersum*	W-C

### 2.2 Detachment of epiphytic biofilm

Epiphytic bacteria attached to submerged macrophytes were collected following the method previously described in published literature (Hempel et al., 2009; He et al., 2012). Briefly, 2 g of each macrophyte with similar growth vigor was selected and rinsed with sterile deionized water three times to remove large particles adhering to the plant surface. Then, the macrophytes were transferred to a sterile 50-mL polyethylene tube containing 40 mL of sodium pyrophosphate (0.1 mol/L Na_4_P_2_O_7_·10H_2_O, NaPPi). The epiphytic bacteria were detached by ultra-sonication for 3 min, followed by 30 min of shaking (225 r/min) and subsequent 3 min of ultra-sonication. The suspensions were filtered onto a 0.22-μm polycarbonate membrane (Millipore, Billerica, MA, USA) to collect the detached epiphytic bacteria and stored at −20°C for DNA extraction.

### 2.3 DNA extraction and PCR amplification

DNA was extracted using the Fast DNA^®^ SPIN Kit for Soil (MP Biomedicals, Irvine, CA, USA) according to the manufacturer’s protocol. The extracted DNA samples were stored at −20°C for further molecular analyses.

PCR was performed to amplify the variable regions of the bacterial 16S rRNA gene (V3–V4) with the primers 341 F (5′-CCTAYGGGRBGCASCAG-3′) and 806R (5′-GGACTACNNGGGTATCTAAT-3′) with sample-identifying barcodes. The PCR mixture with a total volume of 50 μL that included 5 μL of 10× Buffer KOD, 1.5 mM MgSO_4_, 0.2 mM dNTPs, 0.3 μM each of the forward and reverse primers, 1.0 U of KOD DNA Polymerase, 100–300 ng of DNA template, and double-distilled water (ddH_2_O). The reaction was performed as follows: initial incubation step at 94°C for 2 min; followed by 30 cycles of denaturation at 98°C for 10 sec, annealing at 65°C for 30 sec, and extension at 68°C for 30 sec; and final extension at 68°C for 5 min. PCR products were further purified with Agencourt AMPure XP Beads (Beckman Coulter, Brea, CA, USA), and the concentrations were quantified using the ABI StepOnePlus Real-Time PCR System (Life Technologies, Carlsbad, CA, USA). The purified PCR products were combined at equimolar ratios and submitted to Gene Denovo Biotechnology Company (Guangzhou, China) for sequencing on the Illumina HiSeq 2500 PE250 platform.

### 2.4 Sequence analysis

The raw data were filtered and assembled. Raw data obtained after sequencing included dirty reads containing adapters or low-quality bases. Reads with N bases accounting for more than 10% or low quality (containing less than 80% of bases with Q-value > 20) were filtered from the raw data. The filtered reads were then assembled into tags according to the overlap between paired-end reads. The redundant tags were removed from raw tags to obtain unique tags using Mothur (v.1.34.0). The obtained unique tags were then used to calculate the abundance. Operational taxonomic units (OTUs) (97% identity) were clustered using unique tags by Mothur (v.1.34.0) (Schloss et al., 2009). Sequences were taxonomically classified using a 0.5 confidence threshold against the RDP Database (DeSantis et al., 2006). Sequence data have been deposited in the National Center for Biotechnology Information (NCBI) Sequence Read Archive (SRA) database under BioProject PRJNA513113 (sample accession numbers SAMN10696321–SAMN10696330).

### 2.5 Definition of abundant, rare, and conditionally rare taxa

Microbial communities are normally composed of a few abundant and many rare species, and these two subcommunities may have fundamentally different characteristics and ecological roles (Logares et al., 2014). In this study, we defined and classified all OTUs into six categories in accordance with previous studies (Dai et al., 2016; Xue et al., 2018). Briefly, the OTUs with an abundance ≥ 1% in all samples were defined as abundant taxa (ATs); the OTUs with an abundance<0.01% in all samples were defined as rare taxa (RTs); the OTUs with an abundance between 0.01 and 1% in all samples were defined as moderate taxa (MTs); the OTUs with an abundance below 1% in all samples and<0.01% in some samples were defined as conditionally rare taxa (CRTs); the OTUs with abundance ≥0.01% in all samples and ≥1% in some samples but never rare (<0.01%) were defined as conditionally abundant taxa (CATs); OTUs with abundance varying from rare (<0.01%) to abundant (≥1%) were defined as conditionally rare and abundant taxa (CRATs). In this study, we combined ATs, CATs, CRATs, and MTs as ATs to perform further analyses.

### 2.6 Analysis of community diversity

To investigate the diversity of epiphytic bacteria, the α-diversity (including Chao1, ACE, Simpson, and Shannon indices) was calculated for each sample (Kemp and Aller, 2004). To assess sample adequacy, rarefaction curves (3% distance cutoff) were also constructed. Rarefaction curves and α-diversity were performed using Mothur (v.1.34.0). One-way analysis of variance (ANOVA) was performed using SPSS 20.0 (IBM Corp., Armonk, NY, USA) to test the habitat and host plant effects on epiphytic bacterial community composition. The Venn diagram was used to depict the similarities and differences between communities. The Venn diagram was constructed based on the epiphytic bacteria of different submerged macrophytes and different habitats.

First, the Bray–Curtis similarity matrix was calculated to determine the similarity of the epiphytic bacterial community composition between the samples at the OTU level. The cluster analysis based on the Bray–Curtis coefficient was used to investigate the phylogenetic composition differences of microbial communities among 10 samples. Then, the Bray–Curtis similarity matrix was calculated based on samples within the same habitat or the same host plant. The non-metric multidimensional scaling (NMDS) analysis was employed for detecting possible differences in epiphytic bacterial community composition among different habitats and host plants. Analysis of similarities (ANOSIM) was performed to evaluate the significant difference (p< 0.01) between groups. Complete separation is indicated by R = 1, whereas R = 0 indicates no separation (Chen et al., 2017). The NMDS analysis and ANOSIM were performed using the PRIMER v.6.0 package. For this analysis, the data sets ATs, CRTs, and RTs were used.

### 2.7 Niche breadth

Studies have shown that the microbial communities of habitat specialists are more vulnerable to disturbance than those of habitat generalists (Logares et al., 2013; Liu et al., 2017). In this study, to measure habitat specialization, the niche breadth was calculated according to the following formula (Levins, 1968):


$$B_{j}=\frac{1}{\sum_{i=1}^{N}P_{ij}^{2}}$$

where *Bj* is the niche breadth and *Pij* is the percentage of the individuals of species *j* present in habitat *i*. The OTUs with mean relative abundances ≦2 × 10^−5^ were excluded from the analysis, as these taxa may appear to be specialized even if they are not. In this study, there are three types of lake regions with each type representing a habitat.

Species with higher and lower values of niche breadth can be considered generalists and specialists, respectively (Pandit et al., 2009). Compared with habitat specialists, habitat generalists exhibited a more even distribution along a broader range of habitats. Therefore, we classified OTUs as habitat generalists and specialists according to outlier detection.

### 2.8 Predicted functional profiles

Given the need to predict the Kyoto Encyclopedia of Genes and Genomes (KEGG) Orthology (KO) functional profiles of microbial communities, Phylogenetic Investigation of Communities by Reconstruction of Unobserved States (PICRUSt) was developed (Kemp and Aller, 2004). KEGG pathway variability between different habitats or host plants was compared using NMDS or ANOSIM, respectively.

## 3 Result

### 3.1 Taxonomic composition of epiphytic bacterial communities

A total of 515,407 high-quality sequence reads were obtained from all 10 samples and were clustered into 30,435 OTUs based on a 97% similarity level. The sequences that could be annotated to the family level in all 10 samples ranged from 58.51% to 92.00%; therefore, “family” was chosen as the best classification level for the 10 samples (**Supplementary Figure S1**).

**Figure 2:**
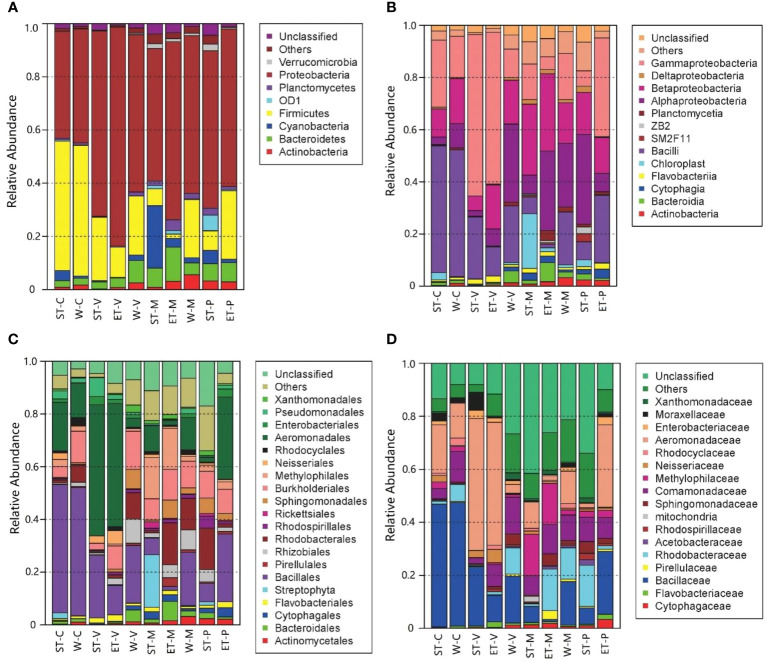
Distribution of epiphytic bacteria in different samples at different taxonomic levels. **(A)** Phylum level, **(B)** class level, **(C)** order level, and **(D)** family level. “Others” refers to the taxa with maximum abundance<2% in any sample. Sequences that could not be classified into any known group were assigned as “Unclassified”.

Significant differences in bacterial abundance among the 10 samples were found in accordance with the bacterial distribution at different taxonomic levels (**Figure 2**). At the phylum level, Proteobacteria were the most dominant group in all 10 samples, which occupied 40.12% (epiphytic bacteria of *C. demersum* in Southern Taihu Lake) to 82.35% (epiphytic bacteria of *V. natans* in Eastern Taihu Lake). The major phyla were Firmicutes (1.56% to 49.05% in each sample), Bacteroidetes (2.42% to 12.84%), Actinobacteria (0.23% to 5.62%), Planctomycetes (0.25% to 4.09%), and Cyanobacteria (0.16% to 23.45%) (**Figure 2A**). Although the abundances of phyla were significantly different between samples, the composition was similar. Furthermore, Alphaproteobacteria, Betaproteobacteria, and Gammaproteobacteria were the dominant classes of Proteobacteria occupying more than 97.9% of Proteobacteria (**Figure 2B**). At the family level, Bacillaceae and Aeromonadaceae belonging to Firmicutes and Proteobacteria, respectively, were dominant in epiphytic bacteria (**Figure 2D**).

### 3.2 Epiphytic bacterial diversity

The coverage of the 10 samples ranged from 88.60% to 98.45%, suggesting that there still may be some microbes remaining undetermined (**Supplementary Table S2**). Rarefaction curves of the 10 samples did not approach saturation at the 0.03 cutoff level, indicating that the amount of sequencing data was not enough to cover all of the sampled species and that a greater bacterial diversity existed in the samples (**Supplementary Figure S2**).

**Figure 3:**
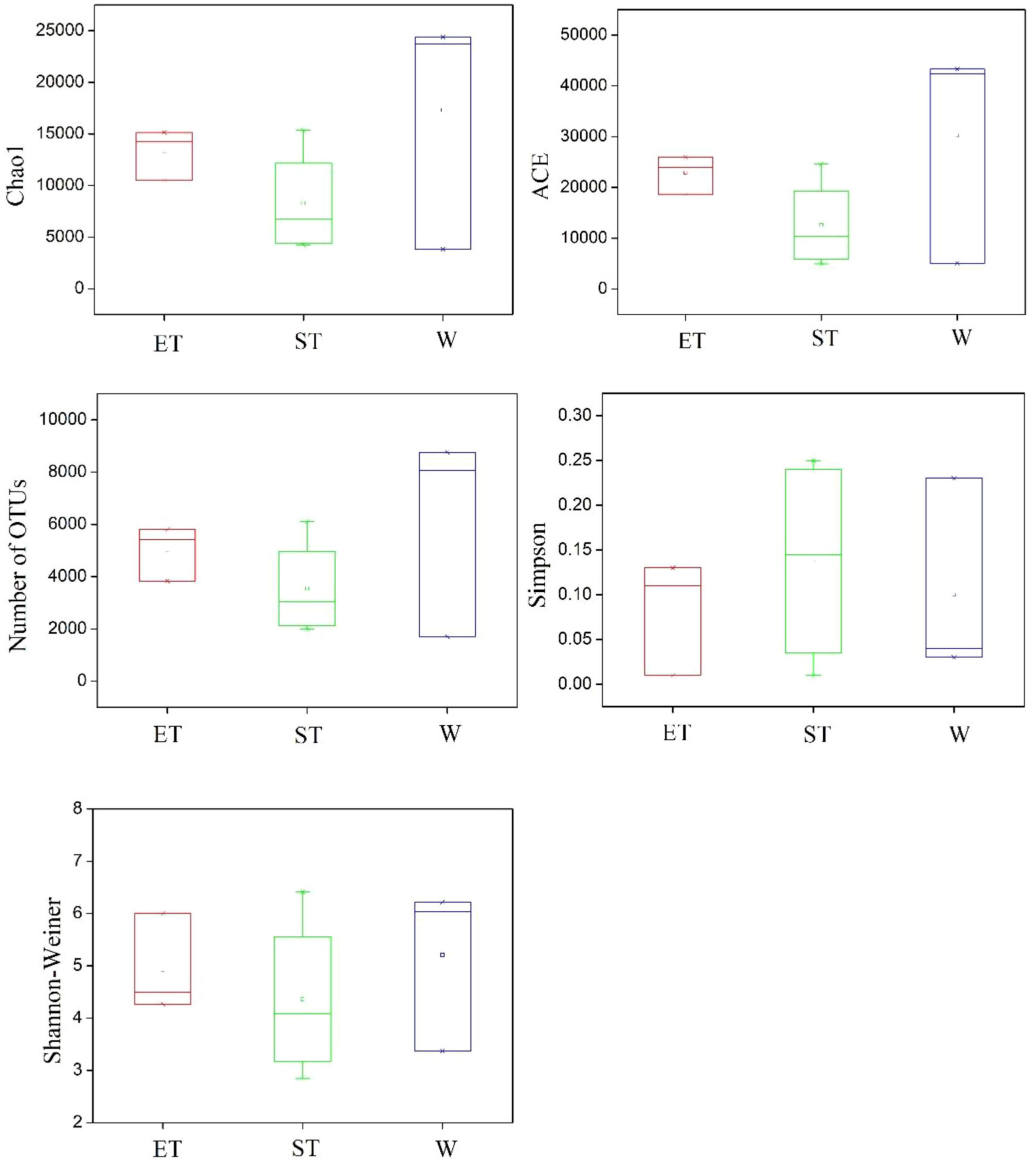
Comparison of alpha-diversity of the epiphytic bacterial communities among three different lake regions. The operational taxonomic units (OTUs) were defined at 97% sequence similarity threshold. The ends of the box represent the 25th and 75th percentiles, the whiskers represent minimum and maximum range, and the center lines represent the median.

The diversity and community richness of epiphytic bacteria were reflected by the Chao1, ACE, Shannon–Wiener, and Simpson indices. The average community richness indices (number of OTUs, ACE, and Chao 1) in Wuli Lake were higher than in Eastern Taihu Lake and Southern Taihu Lake (**Figure 3**). The Shannon–Wiener index ranged from 4.27 to 6.00 (average 4.92) for Eastern Taihu Lake, 2.85 to 6.42 (average 4.36) for Southern Taihu Lake, and 3.37 to 6.22 (average 5.21) for Wuli Lake (**Figure 3**). The average Simpson index in Southern Taihu Lake (0.14) was higher than that in Wuli Lake (0.10) and Eastern Taihu Lake samples (0.08) (**Figure 3**). However, no statistically significant difference (p > 0.05) was observed in diversity indices, suggesting similar species richness and equitability between different lake regions. The one-way ANOVA indicated that neither habitat nor host plant had a significant effect on the alpha-diversity indices (including OTU number, Chao 1, ACE, Shannon–Wiener diversity index, and Simpson diversity index) (**Supplementary Table S3**).

For the bacterial taxa, the distribution characteristics of the three categories identified were as follows: i) 50 (0.16%) OTUs with 290,715 (56.40%) sequences were recognized as ATs, ii) 2,932 (9.63%) OTUs with 175,681 (34.09%) sequences were defined as CRTs, and iii) as many as 27,453 (90.20%) OTUs with 49,011 (9.51%) sequences were classified as RTs (**Supplementary Table S4**).

### 3.3 Variations of epiphytic bacterial community composition

To estimate the patterns of bacterial compositional dissimilarity between samples, the cluster analysis based on the Bray–Curtis coefficient was employed (**Supplementary Figure S3**). Interestingly, among component communities, epiphytic bacterial communities did not group according to host plant or habitat.

**Figure 4:**
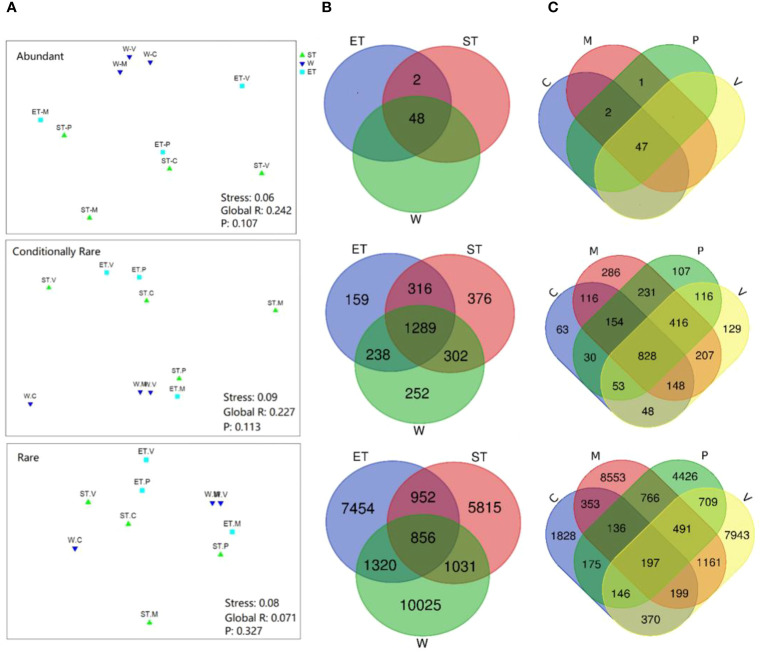
Comparison of beta-diversity of the epiphytic bacterial community among different habitats or host plants. **(A)** Non-metric multidimensional scaling (NMDS) ordination of epiphytic bacterial communities based on the Bray–Curtis dissimilarity. **(B)** Venn diagram showing the shared bacterial operational taxonomic units (OTUs) between three habitats (48 abundant OTUs, 1,289 conditionally rare OTUs, and 856 rare OTUs). **(C)** Venn diagram showing the shared bacterial OTUs between four different types of submerged macrophytes (47 abundant OTUs, 828 conditionally rare OTUs, and 197 rare OTUs).

In terms of relative abundance, our results revealed that all three subcommunities (abundance, conditionally rare, and rare taxa) did not cluster strongly by host plant or habitat, especially the rare subcommunity (**Figure 4A**), which was confirmed by the ANOSIM comparison between epiphytic bacterial subcommunities (**Table 2**). In abundant taxa, Wuli Lake showed a striking separation compared to the other two lake regions, especially Southern Taihu, which suggested that the abundant bacterial subcommunities were vulnerable to environmental variations (**Figure 4A**).

**Table 2:** Results of the ANOSIM and the pairwise analyses comparing epiphytic bacterial communities among different host plants and habitats.

Groups	Abundant taxa	Conditionally rare taxa	Rare taxa
C vs. V	0	0.167	−0.167
C vs. M	0.25	0	0.167
C vs. P	0.5	0	0.25
V vs. M	0.593	0.333	0.111
V vs. P	0	−0.083	−0.25
M vs. P	−0.083	−0.333	−0.417
ST vs. W	0.407*	0.259	0.13
ST vs. ET	−0.019	0.093	0.093
W vs. ET	0.296	0.296	0

The operational taxonomic units (OTUs) were defined at 97% sequence similarity threshold. Values show the R-value, and asterisks denote significant differences at the p< 0.05 level.

ANOSIM, analysis of similarities.

Venn analyses were employed to evaluate the similarity of diversity and community composition between the epiphytic bacterial communities according to lake regions or macrophyte species (**Figures 4B, C**). In the case of the three lake regions, almost all abundant taxa (48 OTUs out of 50 OTUs) were shared among habitats, and most of the unique OTUs belonged to either conditionally rare taxa or rare taxa (**Figure 4B**). A similar pattern was displayed for the epiphytic bacterial community composition according to macrophyte species (**Figure 4C**).

**Figure 5:**
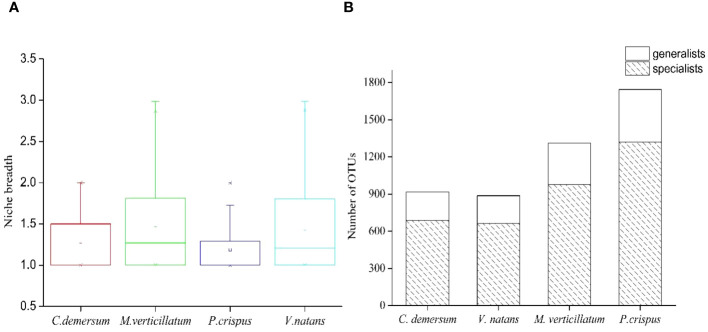
Habitat specialization of different operational taxonomic units (OTUs) based on niche breadth. **(A)** Distribution of niche breadth values of epiphytic bacteria that attached to different host plants. **(B)** The OTU number of generalists and specialists belonged to different host plants.

Microhabitat niche breadth ranged from 1.00 to 2.98 (**Figure 5A**). The niche breadth of epiphytic bacteria displayed variability at different host plants (**Figure 5A**). Mean niche breadth values of epiphytic bacteria were the highest in the host plant *M. verticillatum* (B = 1.45) and *V. natans* (B = 1.44), followed by *C. demersum* (B = 1.25) and *P. crispus* (B = 1.18). For all of the host plants, it could be observed that there were more OTUs identified as habitat specialists than habitat generalists (**Figure 5B**).

### 3.4 Functional annotation and categorization

**Figure 6:**
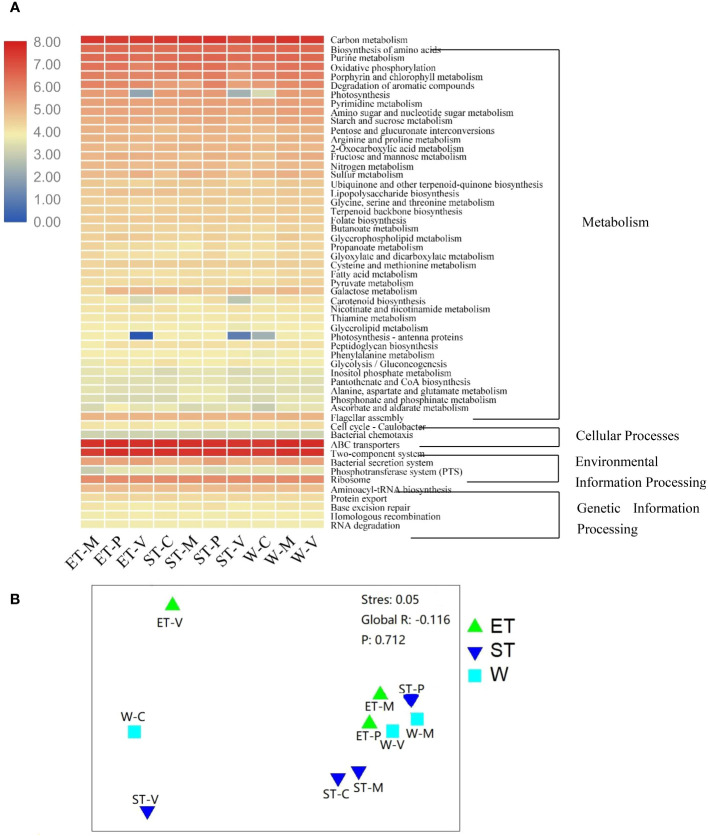
**(A)** Heatmap of differentially abundant Kyoto Encyclopedia of Genes and Genomes (KEGG) pathways among different samples. KEGG pathways with obvious increasing or decreasing trends are displayed in red or blue colors, respectively. **(B)** Non-metric multidimensional scaling (NMDS) analysis based on the calculated Bray–Curtis dissimilarities between the functional gene family abundances of samples.

In order to compare functional differences among different samples, biological functions were predicted and annotated with KEGG pathways. In total, all the OTUs to 139 KEGG pathways were assigned. The most highly represented category was “metabolism pathways”, occupying 66% to 69%, suggesting that the epibiotic bacteria have strong metabolic activity (**Supplementary Table S6** and **Figure 6A**). The “Environmental Information Processing” pathways were also well presented, accounting for 17% to 20% (**Supplementary Table S6** and **Figure 6A**). The predicted functional distribution was not grouped based on either the lake regions or the type of macrophytes (**Figure 6B**), indicating that neither the habitats nor the host plants could significantly influence bacterial functional groups, which is confirmed by the ANOSIM comparisons between differentially abundant KEGG pathways (**Supplementary Table S5**).

## 4 Discussion

Many reports have described the diversity and community composition of epiphytic bacteria on submerged macrophytes. Most studies focused on comparing the composition of the epiphytic bacterial community from either different macrophytes (Ma et al., 2008; He et al., 2012; Gordon-Bradley et al., 2014) or different habitats (Hempel et al., 2008; Levi et al., 2017). However, the current understanding of epiphytic biofilm–macrophyte-specific relationships is far from sufficient. In this study, we aimed to systematically investigate whether different macrophytes or their habitats determine epiphytic bacteria assembly, which will provide clues for understanding the epiphytic biofilm–macrophyte interactions and their roles in freshwater ecosystems.

### 4.1 Effects of different habitats on epiphytic bacterial community

Previous studies have demonstrated that variations in habitats strongly influenced the composition of bacterial communities (Hempel et al., 2008; Levi et al., 2017). Physical habitat characteristics can affect the composition and abundance of epiphytic bacteria by creating microhabitats (Levi et al., 2017). In contrast, our results showed that the composition of the epiphytic bacterial community was not significantly different between different habitats, and the ANOSIM found no significant intra-lake heterogeneity in microbial community structure at different relative abundances (abundant, conditionally rare, and rare taxa) except for the Wuli Lake region and Southern Taihu lake region in abundance taxa (**Table 2**). Cai et al. (2013) obtained similar results when investigating the epiphytic bacterial community structure of *Potamogeton malaianus* Miq. at two lake regions in Taihu Lake. In our study, all the taxa with different relative abundances did not display a separation pattern between groups, whereas the taxa exhibited different responses to the habitats (**Figure 4A**). Typically, the abundant taxa occupy a broad niche and are capable of competitively utilizing a series of limited resources, thus providing driving mechanisms for specific ecological processes (Qin et al., 2022; Zhu et al., 2023). Moreover, the abundant taxa could be more sensitive in response to environmental filtering during colonization (Zhu et al., 2023). In this study, Wuli Lake had a higher degree of eutrophication than the other two lake regions, and the physicochemical properties of lake water differ significantly; therefore, the abundant taxa of Wuli Lake showed a striking separation compared to the other two lake regions. Xian et al. (2018) reported that epiphytic bacteria exhibited a higher diversity in a lower-nutrient environment, which was due to the plant status at different nutrient states. In our experiment, the epibiotic bacteria of macrophytes, which were collected from the least nutrient-impacted region (Eastern Taihu Lake), did not always exhibit higher diversity (**Supplementary Table S2** and **Figure 3**). In brief, the habitats did not have a decisive influence on epiphytic bacterial community composition.

Furthermore, more habitat specialists than generalists were found in the epiphytic bacterial community (**Figure 5B**). Habitat specialists are predicted to be more vulnerable to disturbance than habitat generalists and exhibit specific environmental fitness (Swihart et al., 2003; Michael et al., 2015). Due to the weak ecological tolerance capacity, the specialists were easily affected by environmental disturbance. A recent study showed that habitat specialists showed stronger distance-decay patterns and weaker environmental adaptation than habitat generalists and exhibited higher network complexity and modularity when suffering from environmental stress (Sun et al., 2024). Therefore, when habitats change, the resulting microbial community of epiphytic bacteria is likely to be filled by a series of habitat specialists, which would change the community composition and function.

The reasons for the highly similar epiphytic bacterial community composition between different habitats may be due to the following: i) the epiphytic bacteria not only can absorb nutrients from water but also can use the organic compounds and nutrients secreted by the macrophytes, which may reduce the effects of different habitats on epiphytic bacteria (Cai et al., 2013). ii) The epiphytic bacterial communities may have relatively strong resistance to environmental pressure, thus weakening the environmental fluctuation of the surrounding water (Dong et al., 2014). iii) The epiphytic bacterial communities are also related to the growth state and morphology of the host plant (Crump and Koch, 2008; Lachnit et al., 2011; He et al., 2012).

### 4.2 Effects of plant species on epiphytic bacterial community

Several studies indicated that the different physical or biochemical characteristics of leaves could result in host-specific communities on different plant species (Lachnit et al., 2011; He et al., 2014). In our study, we saw a more complex picture. The ANOSIM suggested that there were no significant differences in community structure at three relative abundances (abundant, conditionally rare, and rare taxa) between different host plants (**Table 2**). However, epiphytic bacterial communities on *C. demersum* from two lake regions (Southern Taihu Lake and Wuli Lake) showed a similar community composition and appeared to be host-specific (**Figure 2** and **Supplementary Figure S3**). Furthermore, the bacterial diversity and bacterial community structure of the four submerged macrophytes were not typical and stable as expected (**Supplementary Table S2**). For instance, the Shannon diversity index showed that *V. natans* had the greatest diversity in Wuli Lake and the lowest diversity in Southern Taihu Lake.

The epiphytic bacteria *M. verticillatum* and *V. natans* displayed a wider niche range than the epiphytic bacteria *C. demersum* and *P. crispus*, suggesting that the epiphytic bacteria *M. verticillatum* and *V. natans* were able to occupy a greater range of habitat types (**Figure 5A**). Hence, species with broader niche breadth had a higher probability of dispersal, thereby resulting in widespread or ubiquitous distribution.

These results indicated that there were sophisticated relationships between epibiotic bacteria and their host plants. To be specific, the epiphytic bacteria on *C. demersum* appear to be host-specific, while epiphytic bacteria on the other three submerged macrophytes were not significantly related to the host plant (**Figure 2** and **Supplementary Figure S3**). The reason may be that the epiphytic bacteria occupy a special ecological niche where they can acquire nutrients from both water body and plant tissues. Hence, the variations in epiphytic bacterial communities may be related to macrophyte species, habitat conditions, or a combination of these factors depending on which factor is overwhelming.

### 4.3 Variation in functional potential of epibiotic bacteria

The predicted functional communities were not separated, as expected, based on habitats or macrophyte species when examining the functions of the epiphytic bacteria (**Figure 6B**). These pathways have been adapted to respond to a wide variety of stimuli. Theoretically, the physicochemical properties of the water body seem to be an essential factor in the functional variation. However, when contrasting the predicted functional variability of epiphytic bacteria, a stable functional distribution was found across different lake regions and macrophyte species (**Figure 6A**, **Supplementary Tables S5**; **S6**). This is congruent with previous research on a marine system, where functional categories were found to be evenly distributed across different zones, while taxonomic compositions varied markedly between subjects (Sunagawa et al., 2015).

In this study, the community composition of all the samples exhibited high variability (**Figure 3**), while the functional categories displayed a stable distribution (**Figure 6**). This suggested that although microbial communities within the same ecosystem may not be identical, their functions may exhibit a high degree of similarity, which is known as “functional redundancy” (Louca et al., 2018; Zhu et al., 2024). These findings were consistent with the redundancy hypothesis, which assumes that there are more than one species sharing common biogeochemical attributes within an ecosystem, thereby conferring ecosystem functions a degree of resilience to disturbance (Naeem, 1998). Moreover, we also observed striking differences among the samples even in the same habitat (**Figures 2** and **4A**), which could not be explained by the neutral model. Therefore, we suggested that the “lottery hypothesis” may be an appropriate model to be able to explain the assembly of epiphytic bacteria. This hypothesis asserts that species with similar ecologies will occupy a niche within an ecosystem based on stochastic recruitment (Brum and Esteves, 2001). Neutral models of community assembly also work on the assumption of ecological equivalence. The difference is that the neutral model assumes the ecological equivalence broadly, while the lottery model makes this assumption for defined groups of species sharing a particular niche (Brum and Esteves, 2001). More specifically, in the lottery model, there is functional redundancy in an ecosystem, and those bacteria that happen to encounter and occupy the surface of macrophytes first are those that will subsequently colonize it.

## 5 Conclusion

Results indicated that the composition of epiphytic bacterial communities was related to macrophyte species, habitat conditions, or a combination of these factors depending on which factor is overwhelming. There are restrictions and limitations with previous studies that emphasized host specificity or environmental heterogeneity in epiphytic bacterial community composition. The assembly strategies of epiphytic bacteria are much more complex than we expected. We believe that there are three main reasons for these inconsistencies: limitations of technology, finite set of samples, and the diversity and complexity of the environment. Our work provided a new perspective for exploring plant–microbe interaction in aquatic systems. This research also provided new evidence for the lottery model as the mechanism that best explains the assembly of epiphytic bacteria.

## Data availability statement

The original contributions presented in the study are included in the article/**Supplementary Material**. Further inquiries can be directed to the corresponding authors.

## Author contributions

HF: Data curation, Investigation, Methodology, Validation, Writing – original draft. ZZ: Software, Writing – review & editing. FY: Investigation, Writing – review & editing. HS: Resources, Writing – review & editing. YW: Writing – review & editing.
