# Supplementary material for: Epiphytic bacterial community composition on four submerged macrophytes in different regions of Taihu Lake
Source: Front Plant Sci. 2024 Jul 25;15:1404718. doi: 10.3389/fpls.2024.1404718 (PMC11306141; doi:10.3389/fpls.2024.1404718)
Supplement: Supplementary file 1 [file DataSheet_1.docx]

**Supplementary Material**

**Epiphytic bacterial community composition on four submerged macrophytes in different regions of Taihu Lake**

* Corresponding author: Hailei Su

E-mail addresses: suhailei666@163.com (H. Su)

No. 8 Dayangfang, Anwai Beiyuan, Chaoyang District, Beijing, 100012, China.

**Number of pages: 7**

**Number of figures: 3**

**Number of tables: 6**

**Table S1.** Environmental variables measured on sampling sites

| Water Environmental Variables | Wuli Lake | Southern Lake Taihu | Eastern Lake Taihu |
| --- | --- | --- | --- |
| Depth (m) | 1.00 | 1.08 | 1.16 |
| Temperature (°C) | 33.64 | 33.37 | 32.42 |
| pH | 9.30 | 8.89 | 9.30 |
| Chlorophyll-a (µg L^-1^) | 5.77 | 3.91 | 2.34 |
| DO (μmol L^–1^) | 10.29 | 6.26 | 7.88 |

Table S2. Estimation of the epiphytic bacterial diversity of 10 samples based on 16S rRNA gene (OTUs were defined as 3% difference in nucleic acid sequence alignment)

| Sample codes | No. of OTUs | Chao 1 | ACE | Shannon (H’) | Simpson (D) | Coverage (%) |
| --- | --- | --- | --- | --- | --- | --- |
| ET-M | 5810 | 15153 | 25922 | 6.00 | 0.01 | 92.86 |
| ET-V | 5408 | 14244 | 23987 | 4.49 | 0.13 | 93.15 |
| ET-P | 3831 | 10515 | 18673 | 4.27 | 0.11 | 94.62 |
| ST-M | 2264 | 4223 | 4932 | 4.69 | 0.06 | 98.11 |
| ST-V | 1981 | 4554 | 6904 | 2.85 | 0.25 | 97.75 |
| ST-P | 6122 | 15347 | 24661 | 6.42 | 0.01 | 92.64 |
| ST-C | 3795 | 8956 | 13739 | 3.48 | 0.23 | 95.48 |
| W-M | 8761 | 24385 | 42319 | 6.03 | 0.04 | 89.42 |
| W-V | 8065 | 23712 | 43311 | 6.22 | 0.03 | 88.60 |
| W-C | 1711 | 3834 | 5070 | 3.37 | 0.23 | 98.45 |

Table S3 Results of one-way ANOVA testing the effects of host plants and habitat on bacterial alpha-diversity.

| Factors | Host Plants | | | Habitat | | |
| --- | --- | --- | --- | --- | --- | --- |
|  | df | F | P | df | F | P |
| Number of OTUs | 3 | 0.484 | 0.706 | 2 | 0.484 | 0.706 |
| ACE | 3 | 0.487 | 0.704 | 2 | 1.586 | 0.270 |
| Chao 1 | 3 | 0.468 | 0.716 | 2 | 1.369 | 0.315 |
| Shannon-Wiener | 3 | 1.396 | 0.332 | 2 | 0.322 | 0.735 |
| Simpson | 3 | 3.410 | 0.094 | 2 | 0.248 | 0.787 |

Note: The operational taxonomic units (OTUs) were defined at 97% sequence similarity threshold.

| Category | Description | OTU number | Sequence number |
| --- | --- | --- | --- |
| Abundant taxa (AT) | Always abundant taxa  Conditionally abundant taxa  Conditionally rare and abundant taxa  Moderate taxa | 1 (0.0033%)  15 (0.049%)  32 (0.11%)  2 (0.0066%) | 78719 (15.27%)  141792 (27.51%)  68088 (13.21)  2116 (0.41%) |
|  |  | 50 (0.16%) | 290715 (56.40%) |
| CRT | Conditionally rare taxa | 2932 (9.63%) | 175681 (34.09%) |
| RT | Rare taxa | 27453 (90.20%) | 49011 (9.51%) |
| Total | All taxa | 30435 (100%) | 515407 (100%) |

**Table S4** General description of abundant, conditionally rare, rare and total taxa data sets at 97% similarity level.

Note: Always abundant taxa (AAT) were defined as the OTUs with a relative abundance ≥1% in all samples; Conditionally abundant taxa (CAT) were defined as the OTUs with a relative abundance ≥1% in some samples but never rare (<0.01%) in any samples; Conditionally rare and abundant taxa (CRAT) were defined as the OTUs with a relative abundance varying from rare (<0.01%) to abundant (≥1%); Moderate taxa (MT) were defined as the OTUs with a relative abundance between 0.01% and 1% in all samples; Conditionally rare taxa (CRT) were defined as the OTUs with a relative abundance <0.01% in some samples but never abundant (≥1%) in any samples; Rare taxa (RT) were defined as the OTUs with a relative abundance <0.01% in all samples; Abundant taxa (AT), including always abundant taxa (AAT), conditionally abundant taxa (CAT), conditionally rare and abundant taxa (CRAT), and moderate taxa (MT).

**Table S5** Analysis of similarity (ANOSIM) results for comparisons between differentially abundant KEGG pathways in different host plants and habitats.

| Groups | Global R | P |
| --- | --- | --- |
| C vs V | -0.5 | 1 |
| C vs M | 0.417 | 0.2 |
| C vs P | 0.5 | 0.333 |
| V vs M | 0.111 | 0.4 |
| V vs P | -0.167 | 0.6 |
| M vs P | -0.333 | 0.9 |
| ST vs W | -0.148 | 0.714 |
| ST vs ET | 0.019 | 0.4 |
| W vs ET | -0.259 | 0.8 |

Note: The operational taxonomic units (OTUs) were defined at 97% sequence similarity threshold.

**Table S6** Summary of the involved KEGG pathways annotation

| **KEGG pathways** | **Numbers of KO** | | | | | | | | | |
| --- | --- | --- | --- | --- | --- | --- | --- | --- | --- | --- |
|  | **ET-M** | **ET-P** | **ET-V** | **ST-C** | **ST-M** | **ST-P** | **ST-V** | **W-C** | **W-M** | **W-V** |
| Metabolism | 1564 | 1570 | 1445 | 1490 | 1521 | 1621 | 1351 | 1402 | 1615 | 1607 |
| Environmental Information Processing | 408 | 468 | 441 | 441 | 426 | 430 | 407 | 386 | 459 | 456 |
| Genetic Information Processing | 190 | 190 | 186 | 189 | 190 | 189 | 184 | 186 | 190 | 188 |
| Cellular Processes | 61 | 59 | 60 | 57 | 60 | 61 | 56 | 58 | 63 | 63 |
| Organismal Systems | 2 | 2 | 2 | 2 | 2 | 2 | 2 | 2 | 2 | 2 |
| Human Diseases | 28 | 41 | 40 | 38 | 37 | 29 | 37 | 36 | 39 | 32 |


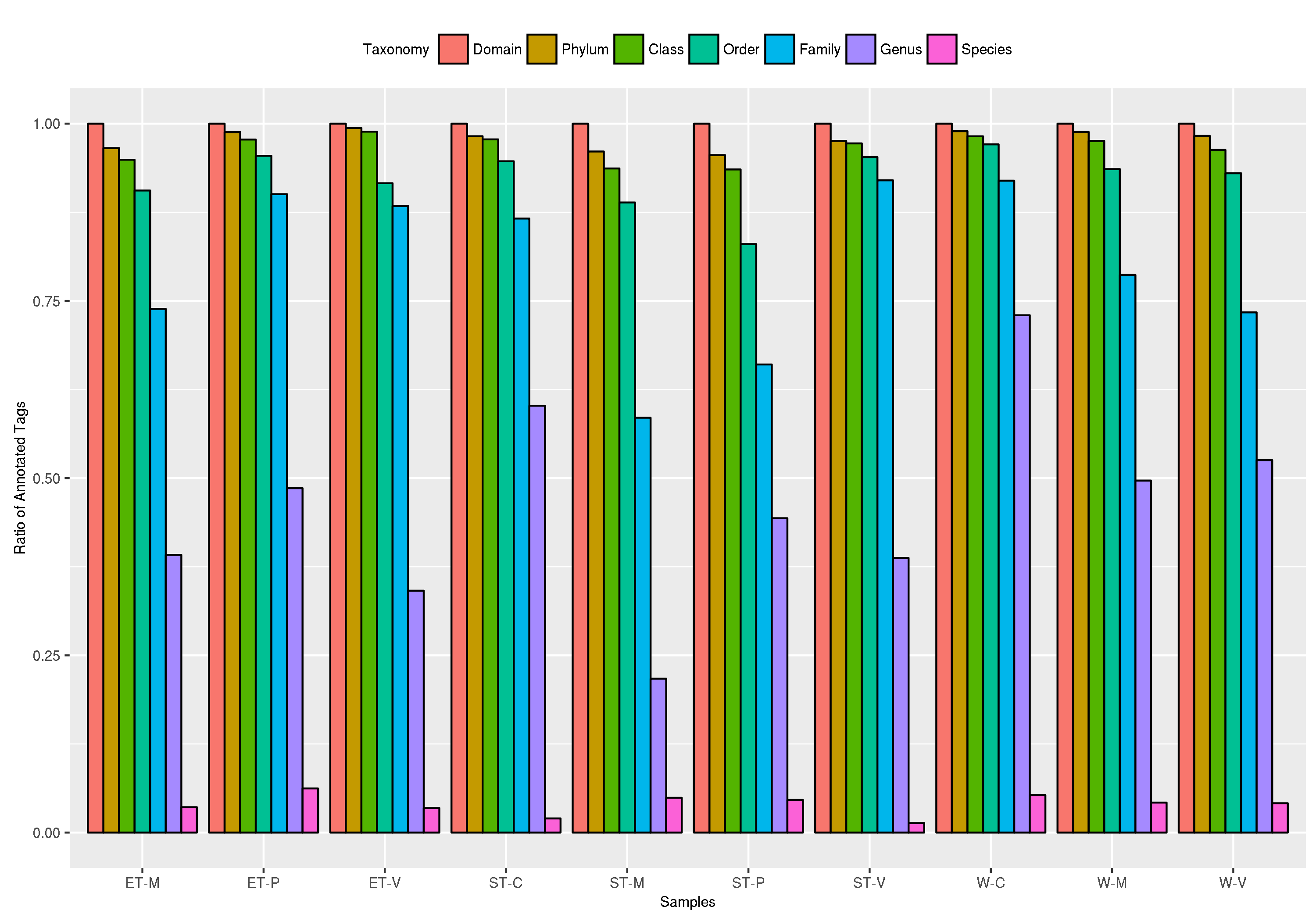


**FIGURE S1** Tags ratio in each classification level.


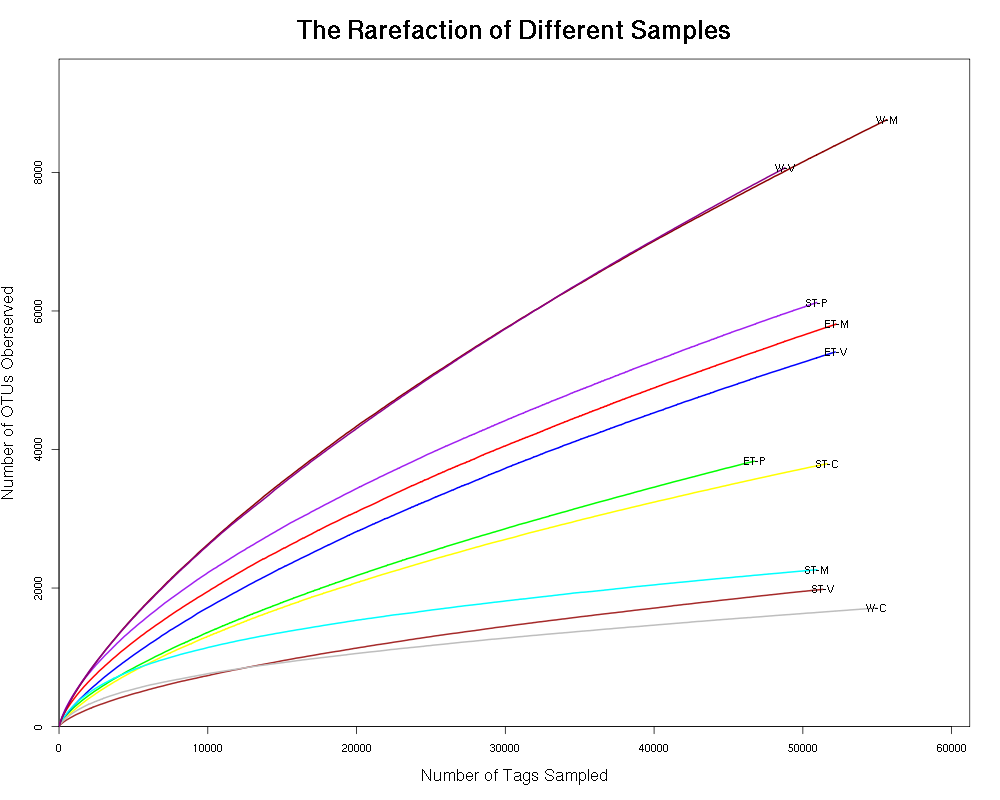


**FIGURE S2** Rarefaction curves of ten samples (at 0.03 dissimilarity level). Sample abbreviations can be found in Table 1.


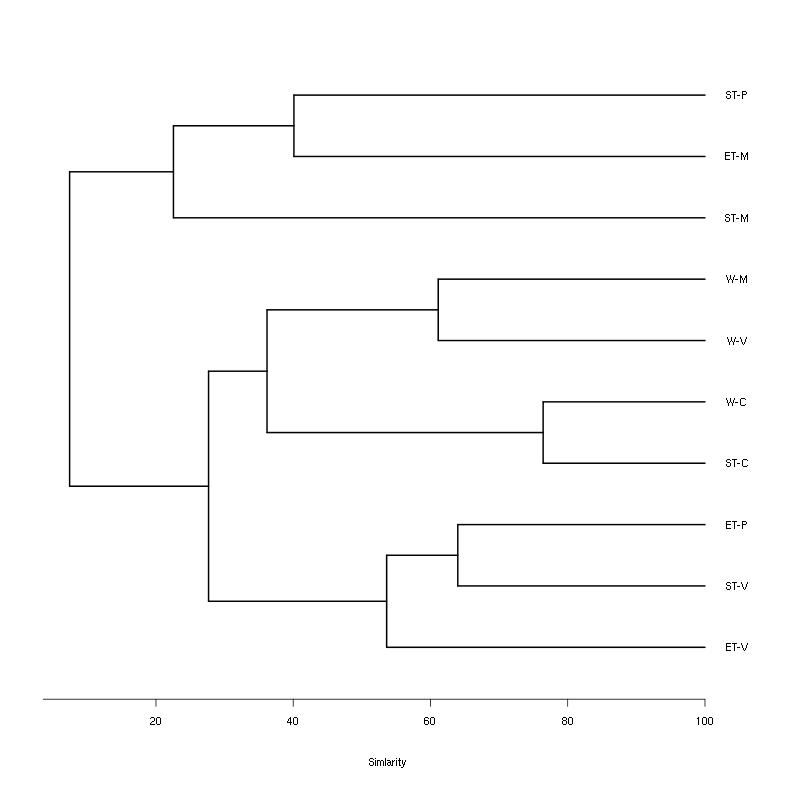


**FIGURE S3** Cluster analysis of epibiotic bacterial community from 10 samples. Sample abbreviations can be found in Table 1.
